# A Comprehensive Review of Polymeric Materials and Additive Manufacturing in Dental Crown Fabrication: State of the Art, Challenges, and Opportunities

**DOI:** 10.3390/polym18060667

**Published:** 2026-03-10

**Authors:** Faisal Khaled Aldawood

**Affiliations:** Department of Industrial Engineering, College of Engineering, University of Bisha, P.O. Box 001, Bisha 67714, Saudi Arabia; faldawood@ub.edu.sa

**Keywords:** 3D printing, additive manufacturing, biocompatibility, dental crowns, photopolymer resins, polymeric materials, prosthodontics

## Abstract

For decades, zirconia- and ceramic-based materials have dominated dental crown fabrication due to their durability and aesthetic appeal. However, a fundamental shift is occurring as polymeric alternatives emerge with notable advantages: better adhesive bonding, versatile aesthetics, lower costs, and a lighter weight. The advances in polymer chemistry and additive manufacturing have significantly impacted prosthodontics, allowing the rapid creation of highly customized, patient-specific restorations with a precision previously impossible (achieved through advanced Computer-Aided Design software and standardized 3D-printing equipment) with traditional methods. This review provides a detailed analysis of 3D-printed polymeric dental crowns from various angles. It explores the materials science behind different polymers, compares manufacturing methods, and evaluates the mechanical performance and biocompatibility. Despite the progress, polymeric materials still fall short of matching the mechanical properties of advanced ceramics, especially in compressive strength and wear resistance. Moreover, there is limited long-term clinical data over five to ten years. The lack of standardized testing protocols complicates cross-study comparisons, and the regulatory pathways for patient-specific 3D-printed devices are still developing, creating uncertainty for manufacturers and clinicians. The future prospective looks promising in many ways such as innovations like four-dimensional printing, where materials respond dynamically to environmental stimuli, which could enable crowns that adapt to changing oral conditions. Nanocomposites with functionalized nanoparticles might enhance mechanical properties while maintaining printability. AI-driven design optimization could automate and improve the crown morphology, occlusal contacts, and fit. Incorporating bioactive materials could turn crowns into active therapeutic devices that promote remineralization and combat bacterial colonization. This review summarizes the current knowledge, highlights the key gaps, and suggests steps toward establishing polymeric 3D-printed crowns as viable long-term alternatives capable of competing with or surpassing traditional ceramic options.

## 1. Introduction

### 1.1. Historical Evolution: From Ancient Gold to Digital Polymers

Restoring damaged teeth is one of humanity’s oldest medical efforts. Archaeological finds in ancient Egyptian tombs show sophisticated gold wire stabilization techniques from over 4500 years ago [1]. Early dental pioneers understood—intuitively, as modern science confirms—that successful tooth restoration needs materials capable of withstanding the oral cavity’s complex biomechanics while integrating seamlessly with living tissues. The use of porcelain in dentistry during 18th-century France marked a pivotal moment [2]. For the first time, restorations could mimic the translucency and color of natural enamel. However, these early porcelain crowns were fragile under biting forces. Dentists then faced a tough choice: excellent aesthetics or mechanical durability. These challenges persisted for two centuries, prompting the ongoing search for materials that combine beauty and strength.

In the mid-20th century, metal–ceramic systems emerged, blending metal’s durability with porcelain’s aesthetic qualities [3]. Porcelain-fused-to-metal (PFM) crowns became the standard for almost fifty years, setting the benchmark for all other materials. Yet, PFM systems had flaws—metal showing through gum margins, porcelain chipping, and difficulties achieving perfect aesthetics in the front teeth. The 1980s’ digital revolution dramatically transformed dentistry with the advent of Computer-Aided Design and Manufacturing (CAD/CAM) [4,5]. These innovations turned crown creation from a manual skill into a precise, repeatable industrial process. Digital impressions replaced messy traditional ones. Computer algorithms designed crowns with exact mathematical accuracy. Milling machines carved restorations from block materials with micrometer precision. Production times went from days to hours, and accuracy improved.

Today, additive manufacturing, building objects layer by layer from digital models, may surpass subtractive milling’s limitations. While milling wastes material and struggles inside complex geometries, 3D printers build efficiently and handle intricate designs easily. Traditional ceramics are rigid; advanced polymers offer adjustable properties. Unlike traditional workflows requiring multiple visits, digital printing allows for same-day restorations. This review explores how additive manufacturing is transforming the fabrication of polymeric dental crowns.

### 1.2. Contemporary Material Options: A Complex Decision Matrix

Modern clinicians face a complex material landscape when selecting crown restorations. This decision tree presents many options, each with different advantages, limitations, and clinical trade-offs [6]. The selection criteria encompass not only traditional considerations, such as tooth location, functional demands, and aesthetic requirements, but also emerging factors including patient financial constraints, environmental sustainability, and digital workflow integration [7]. Feldspathic porcelain, the traditional ceramic workhorse, provides excellent aesthetic properties. Its translucency resembles natural enamel, and skilled ceramists can achieve color matching that renders restorations virtually invisible [8]. However, this aesthetic excellence comes at a mechanical cost. Feldspathic porcelain’s flexural strength typically ranges from 60 to 100 MPa, making it vulnerable to brittle fracture under heavy occlusal loading or impact. Clinicians have learned through decades of clinical experience that feldspathic crowns succeed beautifully in anterior applications with light functional demands but fail catastrophically in high-stress posterior positions. Zirconia emerged in the early 2000s as a transformative material, offering flexural strengths exceeding 900 MPa, nearly ten times that of feldspathic porcelain [9,10]. These exceptional strengths come from zirconia’s unique crystal structure and transformation toughening mechanism. When cracks begin to propagate through zirconia, the material undergoes a stress-induced phase transformation that actually arrests crack growth. These self-healing properties make zirconia very fracture-resistant. Early zirconia formulations sacrificed translucency for strength, limiting the aesthetic applications. However, modern yttria-stabilized zirconia (Y-TZP) and cubic zirconia formulations achieve a translucency approaching lithium disilicate while maintaining impressive mechanical properties. Full metal crowns, typically fabricated from gold alloys or base metal alloys, represent the ultimate in mechanical durability [11]. Gold crowns have demonstrated survival rates exceeding 95% at a 20-year follow-up, a longevity unmatched by any other material. The malleability of gold allows for excellent marginal adaptation, and its biocompatibility is exceptional. Yet, in the current aesthetic-conscious era, the metallic appearance relegates metal crowns primarily to posterior applications where visibility is limited. Even there, many patients reject metal crowns on aesthetic grounds. Porcelain-fused-to-metal crowns attempt to synthesize metal’s strength with porcelain’s aesthetics [12]. This concept is elegant: a cast metal coping provides structural support while porcelain veneering delivers aesthetic appeal. For decades, PFM crowns served as the workhorse of restorative dentistry. However, long-term clinical studies revealed troubling complications. These metal substructures, necessary for strength, create an opacity that compromises aesthetics. Gray shadows appear at gingival margins as gum tissue recedes. The thermal expansion mismatch between metal and porcelain leads to delamination—the porcelain literally pops off the metal framework. When this occurs in the anterior region, the result is both functionally and aesthetically catastrophic. Into this material field come polymers, a diverse class of materials united by their macromolecular structure [13]. Polymer-based crowns offer compelling practical advantages. Processing is straightforward, requiring neither high-temperature furnaces nor expensive crystallization cycles. Repairs can be performed chairside using simple composite additions. Manufacturing costs are lower than ceramics. These materials are lightweight, reducing the stress on supporting structures. Yet, polymers have historically struggled with two critical limitations: mechanical properties inferior to ceramics and questions about long-term stability in the demanding oral environment [14]. The advent of additive manufacturing has renewed interest in polymeric crowns. New material formulations specially designed for 3D printing show improved mechanical properties. Digital workflows enable same-day fabrication impossible with ceramic materials [15]. The ability to print complex internal geometries opens possibilities for stress-optimized designs. Most intriguingly, polymer chemistry’s flexibility suggests pathways to incorporate bioactive components that could make crowns not merely passive restorations but active participants in oral health maintenance.

### 1.3. Manufacturing Fundamental Change: From Subtractive to Additive

Traditional crown fabrication has a long history, with a process refined over centuries but still fundamentally the same in its basic method [6,16]. It begins by taking impressions, either with traditional elastomeric materials or through digital intraoral scanning. These impressions are used to create stone models, which are physical replicas of the patient’s teeth. A skilled technician then sculpts a wax pattern around the prepared tooth, replicating the crown’s shape based on anatomical knowledge and artistic judgment. This wax pattern undergoes lost-wax casting to create a metal coping or serves as a base for pressing or layering ceramic materials. Multiple firings in high-temperature furnaces gradually build the restoration. Finally, the technician stains, glazes, and polishes the crown to achieve the desired aesthetics.

This traditional workflow produces excellent results if done correctly but also has several limitations. The process is labor-intensive, requiring specialized training and manual skill. Each transfer step—from impression to model, wax pattern, and final restoration—introduces potential dimensional errors that can accumulate throughout the process. The entire procedure can take days or weeks, necessitating temporary restorations and multiple patient visits. The material waste is also high, with large amounts of impression material, stone, wax, and investment discarded after each use.

**Figure 1:**
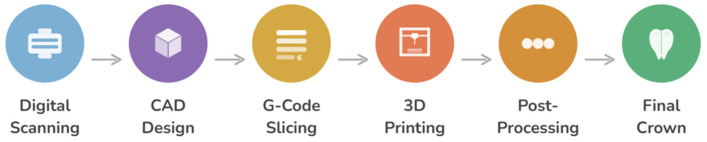
Digital workflow for 3D-printed dental crowns.

Computer-Aided Design and Computer-Aided Manufacturing (CAD/CAM) technology has transformed this process through digitization [17,18]. Intraoral scanners capture tooth preparations as three-dimensional point clouds with sub-50 μm accuracy. Software algorithms automatically generate crown designs based on anatomical libraries and user input. Milling machines carve restorations from pre-made blocks within minutes instead of days. This digital workflow (Figure 1) eliminates many traditional errors—no impression distortion, no stone expansion, no wax distortion. Material properties are more consistent because CAD/CAM blocks are produced through controlled industrial manufacturing rather than hand-layering.

Subtractive manufacturing via milling has become the leading CAD/CAM method [19]. The process is simple in principle: a rotating bur removes material from a block until the final crown shape is achieved. Modern milling systems are highly accurate, operating at 10 μm tolerances. However, subtractive manufacturing has its limitations such as material waste; often, 80–90% of the original block ends up as waste dust. Moreover, complex internal features are difficult or impossible to machine. Moreover, multi-material designs require assembling separately milled parts. Finally, the tool access restrictions limit the design possibilities, and wear on the bur gradually reduces the accuracy over time.

Additive manufacturing, which constructs objects layer by layer from digital designs, employs a different approach [20,21]. Instead of removing material, additive processes build objects directly. This results in minimal material waste since only the material forming the part is used. It can easily create complex internal geometries because the printer forms both external and internal features simultaneously. Multi-material designs are possible through selective deposition. Design freedom increases significantly since tool access is no longer a limiting factor. Notably, the cost per part remains largely unaffected by geometric complexity, reversing traditional manufacturing costs where complex shapes demand higher prices.

For polymeric dental crowns, additive manufacturing offers numerous advantages. Vat photopolymerization systems can achieve resolutions comparable to milling, with layer thicknesses typically between 25–50 μm, and some systems reaching 10 μm. The surface finish quality can match or surpass that of milled restorations. This digital workflow enables same-visit dentistry, with crowns designed and printed while the patient waits. Material properties can be tailored specifically for printing, allowing formulations that are difficult to machine. The technology is highly versatile: the same printer can produce crowns, bridges, dentures, surgical guides, and various prosthetic devices simply by changing materials and design files.

This review explores how additive manufacturing is revolutionizing the fabrication of polymeric crowns. It covers the materials science behind printable polymers, analyzes different printing technologies and their advantages, evaluates the mechanical and biological performance, and highlights key challenges that must be addressed to fully realize this transformative technology.

## 2. Polymer Chemistry and Material Classes for Dental Crowns

### 2.1. Methacrylate-Based Photopolymers: Foundation of Dental 3D Printing

Methacrylate-based resins have become the foundational material class for dental 3D-printing applications [22]. These materials polymerize through free-radical photoinitiation, a mechanism that combines rapid curing kinetics with excellent spatial control. When exposed to light of an appropriate wavelength (typically 365–405 nm), photoinitiator molecules undergo homolytic cleavage to generate free radicals. These highly reactive species attack the carbon–carbon double bonds of methacrylate monomers, initiating chain-growth polymerization. This reaction proceeds rapidly, with gelation occurring within seconds and substantial conversion achieved in under a minute [23]. Rapid curing makes methacrylates ideally suited to vat photopolymerization printing. Each layer solidifies quickly, enabling fast build rates. This reaction is spatially confined to illuminated regions, allowing precise feature resolution. Importantly, methacrylate polymerization proceeds without generating volatile byproducts; the reaction is addition polymerization, simply linking monomers together without eliminating small molecules. This minimizes the internal stress and dimensional changes during curing. Polymethyl methacrylate (PMMA) holds a storied position in dental history, having served as the primary denture base material since the 1930s [24,25]. PMMA’s popularity stems from multiple favorable properties: excellent optical clarity allowing for a natural appearance, good mechanical properties in compression, straightforward processing, and acceptable biocompatibility. For provisional crowns, temporary restorations worn for weeks to months during final crown fabrication, PMMA has long been the material of choice.

Recent research shows that CAD/CAM-fabricated polymethyl methacrylate (PMMA) crowns have better mechanical properties than traditional provisional materials [26], which comes from its procedure. Traditional provisional materials undergo bulk polymerization, where heat from the process can break down polymer chains and cause internal voids. CAD/CAM polymethyl methacrylate (PMMA) blocks are made in controlled industrial environments with optimized curing cycles, resulting in more uniform, higher-molecular-weight polymer networks. When milled into crowns, these blocks produce restorations with flexural strengths of 80–100 MPa, which is higher than the typical 50–70 MPa of conventional provisional materials. However, polymethyl methacrylate (PMMA) has a major limitation that restricts its use to short-term applications: water absorption and the resulting plasticization [27]. PMMA is not fully hydrophobic; the ester bonds in its backbone can form hydrogen bonds with water molecules. Over time, water diffuses into the polymer, acting as a plasticizer that increases molecular mobility and lowers mechanical properties. Studies tracking PMMA properties in artificial saliva show a 20–30% decrease in flexural strength after 3–6 months of immersion. The material also exhibits creep under constant load, slowly deforming over time. These issues explain why PMMA is mainly used for provisional purposes despite its otherwise good properties.

Bis-acryl composite resins are a next step beyond pure PMMA, incorporating inorganic fillers to improve the mechanical properties [28,29]. The term “bis-acryl” refers to bis(methacrylate) monomers, molecules with methacrylate groups at both ends. These difunctional monomers form crosslinked networks instead of linear chains, greatly boosting the mechanical strength and reducing creep. Adding ceramic fillers—typically barium glass or silica in the 40–60 weight percent range—further increases the strength and stiffness while minimizing polymerization shrinkage. Laboratory tests confirm that 3D-printed bis-acryl crowns have enough fracture resistance for provisional use [30]. Fracture load testing, where crowns are placed on simulated tooth preparations and loaded until failure, usually yields values between 800–1200 N. To compare, maximum bite forces in healthy adults range from 400–800 N, with typical chewing forces around 200–400 N. These fracture loads of 800–1200 N provide a comfortable safety margin for provisional crowns. Recent developments in bis-acryl chemistry focus on improving the filler–matrix interface. Silane coupling agents that chemically bond fillers to the polymer matrix significantly enhance the stress transfer efficiency. Nanoscale fillers increase the interfacial area and can reinforce the matrix at the molecular level by restricting the chain mobility. Some experimental formulations incorporate reactive fillers that chemically participate in the polymerization network, creating a true interpenetrating structure rather than merely embedding inert particles.

However, despite these advances, bis-acryl composites still fall short of the requirements for permanent restorations. Long-term wear behavior remains an issue; abrasion from opposing teeth and food particles gradually degrades the surface. The organic matrix is vulnerable to chemical degradation from enzymes in saliva. Color stability over the years remains uncertain, with yellowing and staining commonly observed. Current clinical consensus limits the use of bis-acryl crowns to provisional applications lasting weeks to months.

### 2.2. Composite Resins: Optimizing the Polymer–Filler Balance

Composite resins address the challenge of strength and processability by balancing the filler content with printability [31]. The main idea is straightforward: ceramic fillers improve strength and stiffness, while the polymer matrix provides toughness and ease of processing. The difficulty is increasing filler loadings without making the composite too viscous for printing. Traditional dental composites used for direct restorations contain 70–80% fillers by weight, approaching ceramic-like mechanical properties [32]. These paste composites are placed into cavities and cured in place. However, such high filler levels increase viscosity, hindering 3D printing. Vat photopolymerization requires resins to be fluid enough to form thin layers and separate cleanly, while material jetting demands even lower viscosities for droplet formation [33]. Printable composites usually limit the filler content to 40–55% by weight, maintaining a workable viscosity and offering improved properties over unfilled resins, such as flexural strengths of 120–150 MPa—about 50% higher than pure methacrylate resins—and increased stiffness. The filler size, distribution, and shape significantly influence the performance. Larger particles (1–10 μm) primarily provide mechanical reinforcement through load bearing. Smaller particles (100–500 nm) increase the surface area for bonding and fill the gaps between larger particles, enhancing the packing density. Nanoscale particles (10–100 nm) can penetrate the polymer matrix, affect the chain mobility, and create a nanoscale reinforcement phase. Surface treatment with silane coupling agents is vital for bonding ceramic fillers to organic polymers, enabling effective stress transfer and greatly strengthening the composite. These silane systems are tailored for different filler types and sizes.

Optical properties are critical in dental composites. Larger fillers cause light scattering and opacity, reducing aesthetics. Manufacturers address this by matching refractive indices, using nanoscale fillers that scatter little light, and employing prepolymerized particles to eliminate the index mismatch. Laboratory tests on 3D-printed crowns show promising results but with some limitations [34]. Flexural strengths of 120–140 MPa exceed those of pure polymers but fall short of milled composites (150–180 MPa), mainly due to filler content restrictions. Wear tests reveal that 3D-printed composites wear 2–3 times faster than milled ones, raising concerns about the long-term performance. Wear involves abrasive action from food and opposing teeth, along with surface fatigue from cyclic loading. As the polymer wears away, filler particles can detach, creating a rougher surface that accelerates further wear. Milled composites with a higher filler content resist this degradation better. The main challenge for printable composites is achieving a similar wear resistance at lower filler levels. The degree of conversion—how well carbon double bonds polymerize—affects the mechanical and biological performance of composites [35]. Incomplete conversion leaves unreacted monomers that may leach out, compromising the strength and biocompatibility. Filler particles complicate this process by absorbing and scattering the curing light, hindering complete polymerization. Therefore, post-curing procedures are essential to maximizing polymerization conversion.

### 2.3. High-Performance Polymers: PEEK and the Quest for Permanent Restorations

Polyetheretherketone (PEEK) represents a fundamentally different class of polymer, offering mechanical properties that exceed conventional composite resins, though they remain substantially below ceramic materials such as zirconia (flexural strength 300–1200 MPa) in absolute terms while maintaining several unique advantages [36]. PEEK belongs to the polyaryletherketone (PAEK) family, a high-performance semicrystalline thermoplastic characterized by aromatic rings in its backbone and exceptional thermal and mechanical properties. Unlike methacrylate resins, which polymerize through addition reactions during printing, PEEK is synthesized separately, and then thermoplastically processed: it is heated above its melting point (around 400 °C) for shaping, and then cooled to solidify. PEEK’s mechanical properties are notable for a polymer. Its flexural strength exceeds 150 MPa in the unfilled form and can reach over 200 MPa with fiber or particulate reinforcement. The elastic modulus of 3–4 GPa approximates that of human bone, placing PEEK in a mechanical space between soft polymers and hard ceramics [37]. These intermediate stiffnesses could be advantageous, providing enough strength to withstand masticatory forces while flexing enough to prevent teh stress shielding of supporting structures.

The concept of stress shielding in biomechanics deserves an explanation. When a restoration is significantly stiffer than the supporting tooth structure, it bears most of the load, leaving the adjacent tissues relatively unstressed. Bone and tooth tissues are living and respond to mechanical loading through Wolff’s Law: they adapt by strengthening or weakening as needed. Insufficient loading can cause tissue resorption, while excessively stiff restorations might unintentionally lead to supporting bone loss via stress shielding. PEEK’s intermediate stiffness may help maintain physiological loading patterns.

Beyond mechanical properties, PEEK offers exceptional chemical stability. This polymer is essentially inert in the oral environment, resistant to typical oral fluids, foods, and pharmaceuticals. Long-term implantation studies in orthopedics, where PEEK is used for spinal cages and joint replacements, show no degradation even after years of in vivo exposure. This chemical inertness ensures property stability over time and addresses a key limitation of methacrylate resins. The research confirms that PEEK crowns can withstand typical posterior loading [14]. A finite element analysis of masticatory forces on PEEK crowns predicts stress distributions well within the material’s strength limits. Experimental tests validate these predictions: PEEK crowns subjected to cyclic loading, simulating years of mastication, show minimal property degradation. Notably, the fatigue resistance of PEEK is impressive, with fatigue limits around 50% of the ultimate tensile strength, compared to 20–30% for many polymers. However, several significant challenges limit the current clinical use of PEEK. Its high melting temperature (around 400 °C) requires specialized processing equipment incompatible with most dental 3D printers [38]. Conventional vat photopolymerization is not possible because PEEK is not photocurable. Fused filament fabrication (FFF) could work, theoretically, but demands heated chambers and nozzles capable of operating above 400 °C, well beyond standard FFF equipment. Some manufacturers have developed high-temperature 3D printers specifically for PEEK, but these are costly specialty devices.

An even more problematic issue is PEEK’s poor adhesion to the tooth structure and dental cements. The same chemical inertness that provides stability also prevents bonding. Its aromatic hydrocarbon surface is highly hydrophobic and low-energy, lacking the functional groups necessary for chemical bonding. As a result, crowns must mainly rely on mechanical retention rather than adhesive bonding—a disadvantage compared to ceramics, which bond reliably with resin cements. Extensive research has focused on PEEK surface modification to improve adhesion [39]. Strategies include plasma treatment to create reactive surface groups, acid etching to increase surface roughness and energy, and coating with adhesion—promoting intermediates. Sulfuric acid etching, in particular, has shown promise in creating a rough surface topology and adding sulfonate groups that enhance wettability and enable chemical bonding. Newer approaches involve silane primers specifically developed for PEEK and atmospheric plasma treatments, which avoid harsh chemical processing.

Despite these challenges, PEEK’s potential keeps it a focus for researchers. These materials offer the ideal combination: mechanical properties below ceramics, biocompatibility comparable or superior to titanium, chemical stability for long-term performance, and processing flexibility for complex geometries. Solving the processing and adhesion issues could position PEEK as a leading material for permanent polymeric crowns.

### 2.4. Hybrid Materials: Engineering Interpenetrating Networks

Polymer-infiltrated ceramic networks (PICNs) are an innovative way to create materials with better properties than either component alone [40]. It is important to note upfront that PICNs are not currently 3D-printable through direct additive manufacturing; their fabrication requires conventional multi-step ceramic processing. Within this review’s scope, PICNs are therefore best understood as an emerging hybrid concept that integrates indirectly with additive manufacturing workflows, rather than as a direct AM output. Future AM strategies targeting PICN-like structures are discussed at the end of this section. The idea is simple: make a porous ceramic scaffold with interconnected pores, then fill it with a polymer. This results in a true interpenetrating network where neither phase merely disperses in the other; instead, both form continuous, interconnected phases throughout the material. The manufacturing process starts with ceramic powder pressed into green bodies with a controlled porosity (usually 25–35% by volume). Partial sintering fuses the ceramic particles, giving the material mechanical strength while keeping open pore channels. This porous scaffold is then vacuum-infiltrated with the methacrylate monomer, which is later polymerized in place. The final material contains approximately 75% ceramic and 25% polymer by volume, with both phases forming interpenetrating three-dimensional networks [41].

This microstructure results in impressive mechanical performance [42]. The ceramic networks provide strength and stiffness, with flexural strengths exceeding 150 MPa, surpassing that of any pure polymer. The polymer network helps absorb cracks and toughen the material. When cracks try to spread through the ceramic, they encounter polymer-filled gaps that absorb energy and deflect the crack path, significantly improving the fracture toughness compared to pure ceramics. Some PICN materials achieve toughness values 2–3 times higher than the ceramic alone. The elastic modulus of PICNs (around 30–40 GPa) falls between that of pure polymers (2–5 GPa) and ceramics (60–100 GPa). This intermediate stiffness may provide biomechanical advantages, offering enough strength for load bearing while allowing enough flex to distribute stresses more evenly across the tooth structure. Clinically, this could lead to fewer crown fractures and the better preservation of the remaining tooth structure.

Machinability is another advantage of PICNs. These materials are easier to mill than ceramics because the polymer phase lubricates cutting and prevents catastrophic chip formation. Wear on cutting tools (burs) is reduced, machining forces are lower, and edge chipping—which is common when milling pure ceramics—is largely eliminated. These processing has the benefit of lower manufacturing costs and improves precision. However, a significant challenge of using PICNs in 3D printing is their multi-step fabrication process. Creating the porous ceramic scaffold requires conventional ceramic processing, which includes forming a green body and controlled sintering. The infiltration step requires vacuum equipment to ensure complete polymer penetration. After infiltration, polymerization needs additional curing. This complexity makes PICNs less suitable for direct 3D printing. Some researchers are exploring indirect methods, such as printing a polymer–ceramic composite, burning out the polymer to create porosity, and then re-infiltrating with another polymer. But these methods are still experimental and labor-intensive.

Despite these processing challenges, PICN materials show that microstructural engineering can overcome the limitations of individual components. The concept of interpenetrating networks, where complementary materials mutually reinforce each other through close three-dimensional mixing, offers a powerful design approach. Future developments may enable the direct 3D printing of PICN-like structures using multi-material printing or by printing precursors that transform into interpenetrating networks during post-processing.

**Figure 2:**
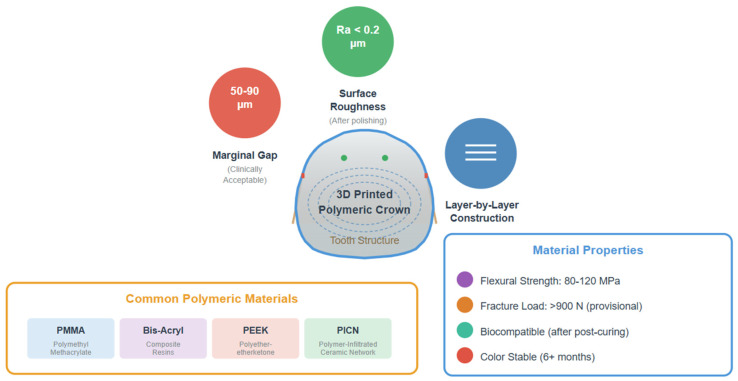
Structure and key properties of 3D-printed polymeric dental crown.

A comparative analysis of polymeric material classes utilized in the fabrication of 3D-printed dental crowns is presented in Table 1. Furthermore, the structural characteristics and key physicochemical properties of 3D-printed polymeric dental crowns are schematically illustrated in Figure 2.

## 3. Additive Manufacturing Technologies: Principles, Capabilities, and Limitations

### 3.1. Vat Photopolymerization: The Premier Technology for Dental Applications

Vat photopolymerization includes a group of related technologies—stereolithography (SLA), digital light processing (DLP), and liquid crystal display (LCD) printing—that share a core operating principle but vary in implementation details [23]. All involve selectively curing liquid photopolymer resin in a vat to build three-dimensional objects layer by layer. This method has become the leading additive manufacturing technology for dental applications because it offers a balanced combination of resolution, surface finish, material properties, and build speed [43]. The basic process remains consistent across these technologies. A build platform is positioned just below the surface of the liquid resin (or just above in inverted setups). Light of an appropriate wavelength selectively cures a thin resin layer that corresponds to a cross-section of the part. The platform then moves vertically by one layer thickness—usually 25 to 100 μm—and new liquid resin flows over the previous layer. The next cross-section is then cured, bonding to the layer below. This sequence repeats hundreds or thousands of times until the full part is completed.

#### 3.1.1. Stereolithography: Precision Through Laser Scanning

Stereolithography, the pioneering 3D-printing technology developed in the 1980s, uses a focused laser beam to selectively cure photopolymer resin [44]. A UV laser (usually 355 nm) is guided by galvanometer mirrors that enable high-speed X–Y positioning. This laser traces the boundary and interior of each layer, with the exposure time and scanning speed calibrated to achieve the proper cure depth. Modern SLA systems achieve spot sizes as small as 50 μm, allowing for fine feature resolution. SLA’s laser-scanning method offers several advantages. These focused lasers deliver a high energy density, enabling deep cure depths that enhance the interlayer bonding strength. These scanning strategies can be tailored for different geometries, with boundaries traced at slower speeds for higher precision, while interior regions are filled quickly with hatching patterns. This technology supports a variety of photopolymer formulations, from low-viscosity liquids to moderately filled systems, because the laser power can be adjusted to match different optical properties.

Clinical studies confirm that crowns made with SLA achieve excellent dimensional accuracy [45]. Research using coordinate measuring systems or optical scans has shown that the total crown dimensional deviations are typically below 50 μm, comfortably within the 120 μm threshold considered clinically acceptable for a marginal fit. Importantly, the accuracy remains consistent across the build volume because laser positioning systems maintain their precision regardless of location. This consistency allows for the batch production of multiple crowns with a uniform quality. The layer-by-layer approach enables internal geometries that are impossible with subtractive manufacturing [46]. Imagine a crown with internal stress-relief channels or a design with undercut features to improve retention. Traditional milling cannot produce such features because cutting tools cannot access trapped internal spaces. But 3D printing builds inside and outside simultaneously, limited only by the requirement that all features be self-supporting or have removable support structures.

SLA’s main limitation is building speed; since the laser must scan each layer sequentially, build time directly correlates with the cross-sectional area. Large parts or the batch production of many parts can take hours. These lasers also need to pause periodically while the resin surface stabilizes after platform movement, adding further time. For single crowns, build times of 15–30 min are typical and acceptable for chairside fabrication, though slower than milling (usually 10–15 min).

#### 3.1.2. Digital Light Processing: Parallel Processing for Speed

Digital Light Processing (DLP) addresses SLA’s speed limitation through a fundamentally different method: projecting entire layer patterns at once instead of scanning point by point [47]. A DLP system uses a digital micromirror device (DMD), which is a chip with millions of tiny mirrors that can tilt individually to reflect light toward or away from the resin. By coordinating these mirrors, the DMD functions as a dynamic mask, projecting arbitrary 2D patterns. This parallel exposure method greatly speeds up printing. While the SLA build time depends on the layer’s cross-sectional area (more area means more scanning), the DLP layer time remains nearly constant regardless of the number of parts or their complexity. Exposing a single crown takes about the same time as exposing five crowns at once. These features make DLP especially suitable for batch production; dental labs can print entire cases in the same time SLA would take to print just a single crown. The research comparing DLP to SLA shows a similar dimensional accuracy but with significantly shorter build times [48]. A comprehensive study evaluating both for crown fabrication found average dimensional deviations below 10 μm, which is clinically insignificant. However, DLP completed the builds 40% faster. For labs that produce multiple crowns daily, this speed advantage directly increases the throughput and lowers the per-unit costs. DLP’s parallel processing offers unique opportunities for optimization [49]; for example, a dental lab with orders for 10 crowns and 5 bridges can arrange all parts on the build platform and print them simultaneously, rather than sequentially. The total build time equals that of the tallest part, around 20–25 min. By contrast, SLA would require sequential printing, multiplying the total time by the number of parts. This parallelization fundamentally changes the manufacturing economics, making batch production far more efficient.

However, DLP also has its challenges, like the fact that most commercial DLP systems use UV LEDs instead of lasers as the light source. While more affordable and compact, LEDs provide lower-intensity and less-uniform illumination across the build area, which can cause variations in the cure depth and material properties depending on the part’s location on the platform. Careful calibration and compensation algorithms are necessary in order to ensure consistent quality. The resolution in DLP systems depends on the projector’s native pixel resolution and optical magnification. A projector with 1920 × 1080 pixels over a 100 mm × 75 mm build area results in about 50 μm pixels, suitable for crowns but limited for fine details. Higher-resolution systems require smaller build areas or higher-resolution projectors, both increasing costs. Some manufacturers address this with multi-resolution approaches, using different projectors for various applications.

#### 3.1.3. LCD-Based Systems: Democratizing Dental 3D Printing

LCD-based 3D printing is the latest step in vat photopolymerization, utilizing liquid crystal displays as dynamic masks [50]. This technology closely resembles DLP, projecting entire layer patterns at once, but it uses LCD panels instead of DMD chips. An LCD panel either blocks or allows UV light from a backlight LED array, forming the layer pattern. When transparent, light passes through to cure the resin; when opaque, curing is blocked. Unlike DMD chips, which are expensive specialty parts, LCD panels are mass-produced items found in smartphones, tablets, and monitors. This makes LCD 3D printers much cheaper than comparable DLP systems, often by a factor of 5 to 10. For dental practices or small labs with tight budgets, LCD printing offers access to additive manufacturing that was previously too costly.

Recent clinical studies show LCD-printed provisional crowns achieve the necessary accuracy and fit [51]. One study of 30 LCD-printed crowns reported marginal gaps averaging 65 μm with a standard deviation of 15 μm, well within clinical requirements. The internal fit was also acceptable, with gap measurements ranging from 80 to 120 μm in different areas. Patients reported high satisfaction, with no issues related to comfort or function during the provisional phase.

However, LCD technology has some limitations in UV transmission efficiency. LCD panels need to absorb or reflect light to create dark areas, which wastes photons that could otherwise cure the resin. The typical LCD transmission efficiency is only 5–10%, meaning 90–95% of the backlight’s light is lost. This requires very bright LED arrays and longer exposure times compared to DLP systems. Although the build speeds are usually slower than DLP, they are still faster than the laser-scanning SLA. Another issue is the lifespan of the LCD panel. Continuous high-intensity UV exposure gradually damages the liquid crystals and polarizing films, reducing transmission over time. Most LCD printers need a panel replacement after 500 to 2000 h of use. While replacement panels are inexpensive, this maintenance adds complexity. Some manufacturers have developed protective films and wavelength-optimized backlights to prolong panel life.

Despite these challenges, LCD printing is making dental additive manufacturing more accessible. Practices that could never justify spending over $50,000 on SLA or DLP systems can now access similar capabilities for under $5000. Lower costs are fueling wider adoption and enabling new experimentation with digital workflows that were previously financially out of reach for smaller practices.

### 3.2. Material Jetting: Multi-Material Possibilities and Precision

Material jetting represents a fundamentally different additive manufacturing approach, building objects by selectively depositing droplets of photocurable material [52]. This technology is similar to inkjet printing scaled up to three dimensions, with printheads containing hundreds of tiny nozzles that jet droplets onto a build platform, where UV lamps immediately cure them. By controlling which nozzles fire and in what sequence, complex 3D geometries are constructed droplet by droplet (the defining feature of this technology is multi-material printing) [53]. Professional material-jetting systems include multiple printheads loaded with different materials, such as a rigid photopolymer, a flexible photopolymer, support material, and color tints. By coordinating the droplet deposition from multiple heads, objects with varying properties can be created within a single build. For example, a crown with a rigid occlusal surface transitioning to a more flexible cervical region can be printed as a single piece without assembly.

Material jetting offers exceptional resolution and surface quality. Droplet sizes as small as 15 μm allow for fine features, and the droplet-stacking process naturally produces smooth surfaces without the layer lines typical of other technologies. The parts often require no post-processing beyond support removal. For applications requiring aesthetic quality, such as anterior crowns, the improved surface finish is particularly valuable. The technology also supports full-color printing by jetting cyan, magenta, yellow, and clear materials in controlled ratios: any color can be produced at the voxel level. This capability has enabled the creation of highly realistic diagnostic models and surgical guides with color-coded information. For crowns, full-color printing could produce natural-looking restorations without external staining, although the current material properties limit this use to models rather than functional prostheses.

However, material jetting faces several significant limitations that hinder its adoption in dentistry. These specialized materials are proprietary and costly, often 10–20 times more expensive than generic photopolymer resins used in vat systems. The printheads are complex and precise devices prone to clogging, requiring regular maintenance. Most importantly, the materials are optimized for printability (low viscosity, and consistent droplet formation) rather than mechanical strength, resulting in parts that are weaker than those produced by vat photopolymerization. Moreover, the equipment costs are a barrier for most dental practices [54]. Professional material-jetting systems capable of producing dental-quality prints usually cost between $150,000 and $500,000, an order of magnitude higher than the SLA/DLP/LCD alternatives. This positions material jetting as a specialized technology for specific applications (like multi-material surgical guides) rather than a general solution for crown fabrication. Unless the material costs decrease and the equipment becomes more accessible, material jetting will likely remain a niche technology in dentistry.

### 3.3. Fused Filament Fabrication: Low-Cost Access with Limitations

Fused Filament Fabrication (FFF), also called Fused Deposition Modeling (FDM), provides the easiest entry point into 3D printing [55]. The technology is mechanically simple: a thermoplastic filament is heated in a nozzle and extruded onto a build platform or onto an earlier layer. As the molten material cools, it hardens and bonds to the neighboring material. Nozzles move in X–Y while the platform moves in Z, creating layers to form 3D objects. FFF’s main benefit is its accessibility; for example, the consumer-grade FFF printers can cost as little as $200–$500, while dental-grade systems range from $2000 to $5000. Filaments are affordable, typically costing $20–$50 per kilogram. The technology is mechanically durable, with few complex parts, making it reliable and simple to maintain. This combination of low cost and simplicity has made FFF the most popular 3D-printing method worldwide, with millions of hobbyists and professionals using it.

However, FFF has significant limitations for dental crown production [20]. The resolution is limited by the nozzle diameter (usually 400–600 μm) and layer height (usually 100–200 μm), which are much coarser than vat photopolymerization. The layer-by-layer process leaves visible lines that need extensive finishing to remove. The surface quality is poor, with a texture and roughness unsuitable for intraoral use without major post-processing. Moreover, the material choices for FFF are limited by the processing needs. The materials must be melt-processable (melt without degrading), resist warping during cooling, and have enough melt viscosity for consistent extrusion. Common FFF materials include PLA (polylactic acid), ABS (acrylonitrile–butadiene–styrene), and PETG (polyethylene terephthalate glycol). None of these meet the strict requirements for permanent dental use because of the inadequate mechanical strength, limited biocompatibility, or poor water resistance. Some manufacturers have produced medical-grade filaments designed for dental use [56]. These often use polycarbonate or PEKK (polyetherketoneketone) as the base polymers, with additives for better processing and biocompatibility. Nevertheless, even with improved materials, FFF-printed crowns tend to have weaker mechanical properties compared to SLA or DLP parts, because of the layer-by-layer build. These parts exhibit anisotropic properties—being weaker between layers—and often contain porosity from the incomplete bonding between strands.

Today, dental FFF is mainly used for study models, surgical guides, and orthodontic models where high precision is not critical [20]. For these purposes, FFF’s low cost and quick prototyping offer clear benefits. Recent work has also explored the multiresponse optimization of FFF process parameters to improve the mechanical output for such applications. But, for crown fabrication requiring sub-100 μm accuracy, smooth surface finishes, and uniform mechanical properties, vat photopolymerization remains the better choice despite the higher equipment costs.

**Figure 3:**
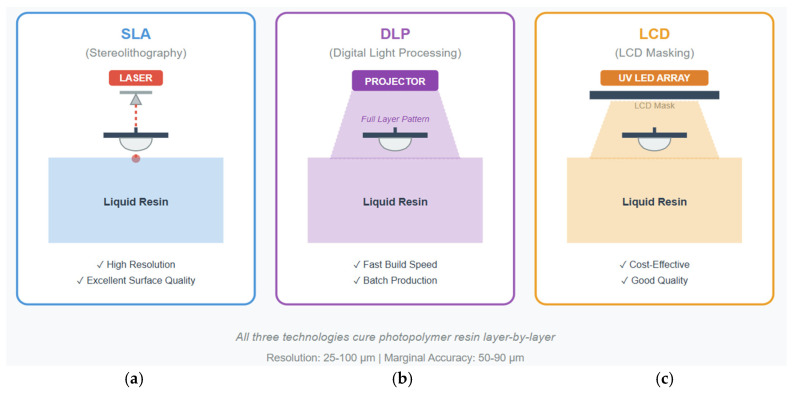
Schematic comparison of vat photopolymerization technologies: (**a**) stereolithography laser scanning, (**b**) digital light processing projection, and (**c**) LCD-based masked projection.

A comprehensive comparison of additive manufacturing technologies used to fabricate dental crowns is presented in Table 2. Additionally, a schematic comparison of the principal vat photopolymerization technologies is illustrated in Figure 3.

## 4. Mechanical and Functional Performance: Meeting Clinical Demands

### 4.1. Fundamental Mechanical Requirements for Dental Crowns

Dental crowns must meet strict mechanical requirements to survive the harsh oral environment [57]. Unlike many engineered structures that experience relatively consistent, predictable loading, crowns are subjected to highly variable, multidirectional forces thousands of times daily. During mastication, forces exceed 500–800 N in the posterior region, applied at varying angles depending on the food properties and jaw movement. These forces occur cyclically; meals produce thousands of loading cycles daily, adding up to millions over years of use. Besides mastication, crowns must withstand parafunctional habits like bruxism (teeth grinding), which can generate forces over 1000 N during sleep. They must also resist impacts from accidentally biting hard objects and maintain dimensional stability despite temperature fluctuations from hot and cold foods spanning 0–60 °C. The key mechanical properties for crown materials include flexural strength (resistance to bending failure), compressive strength (resistance to crushing), elastic modulus (stiffness), fracture toughness (resistance to crack growth), and fatigue endurance (ability to withstand repeated loading). Aside from these basic properties, wear resistance is essential for long-term function; gradual surface loss from abrasion caused by food particles and opposing dentition determines the overall lifespan of many restorations.

#### 4.1.1. Flexural Strength and Fracture Resistance

Flexural strength testing using three-point or four-point bending provides the essential mechanical characterization of dental materials [58,59]. These tests apply a load to a rectangular specimen supported at two points, measuring the force needed to cause fracture. Calculations based on beam theory produce flexural strength, usually expressed in megapascals (MPa). For dental polymers, this test is especially relevant because crown loading during mastication involves significant bending moments, particularly in posterior teeth where cusps experience complex stress patterns. Contemporary 3D-printed polymers show flexural strengths ranging from 80 to 150 MPa, depending on material formulation and processing conditions. Unfilled methacrylate resins typically reach 80–100 MPa, suitable for provisional uses but only marginal for permanent restorations. Composites with a 40–50% filler content achieve 120–140 MPa, approaching the lower range of traditional dental ceramics. PEEK and other high-performance polymers can exceed 150 MPa even without reinforcement. Despite this, the flexural strength measured on standardized test specimens only partly predicts the clinical crown performance. The shape of actual crowns, with their complex occlusal surfaces, varying wall thickness, and stress concentrations at the margins, results in stress distributions far more complicated than simple beam-bending models. Finite element analysis studies that model realistic crown geometries and loading conditions show that peak stresses can reach two to three times the nominal stress calculated from basic force/area methods. Stress concentrations are especially common at the occlusal central fossa, along inclined cuspal surfaces, and at the crown–tooth interface.

Fracture resistance testing with crown-shaped specimens offers more clinically relevant data [22]. These tests involve fabricating crown-shaped specimens, cementing them onto simulated tooth preparations (either extracted teeth or resin analogs), and loading them until failure using a spherical indenter on the occlusal surface. This approach better captures the complex geometry and support conditions found in actual clinical situations. Such tests reveal that properly processed 3D-printed crowns can withstand fracture loads of 800–1200 N, considerably above typical masticatory forces of 200–400 N in the posterior region [22]. However, these values are still significantly below the load that ceramic crowns can endure, which is usually 1500–2500 N. The safety factor, defined as the ratio of the fracture load to the expected service load, is around 2–3 times for polymeric crowns compared to 4–6 times for ceramics. While a safety factor of 2–3 is generally acceptable for engineering purposes, the unpredictable nature of oral forces and the potential need for crown replacement suggest that higher safety margins are preferable.

#### 4.1.2. Elastic Modulus and Stress Distribution

The elastic modulus, a measure of material stiffness defined as stress per unit strain, significantly affects how crowns distribute loads to the supporting structures. Materials with very high modulus values (such as zirconia at 200–220 GPa) tend to concentrate stresses within the crown itself, possibly protecting the underlying tooth structure from physiological loading. Conversely, materials with very low modulus values (like unfilled polymers at 2–4 GPa) deform too much, spreading loads more broadly but risking occlusal settling over time. The polymeric crown materials fall somewhere in between, like Unfilled methacrylates, which have moduli of 2.4–3.4 GPa, composite formulations with filler loading reaching 8–15 GPa, and PEEK and similar high-performance polymers achieving 3–4 GPa. These values are lower than those of ceramics (60–220 GPa, depending on type), and are also substantially lower than natural dentin (15–25 GPa, depending on the hydration state and testing method). Polymeric materials thus exhibit elastic moduli approximately 12–20% of dentin’s modulus, and only 1.5–3% of zirconia’s modulus. To clarify explicitly, unfilled methacrylate resins exhibit elastic moduli of only 2–3 GPa, and even high-performance PEEK falls in the 3–5 GPa range. Both values are substantially below natural dentin (approximately 15–25 GPa). Therefore, polymeric crown materials are mechanically softer than dentin, not stiffer—a key distinction with significant biomechanical implications. The implications of these differences are complex.

A finite element analysis of the stress distribution in crowned teeth suggests that materials with intermediate moduli may offer biomechanical benefits. When the crown’s modulus closely matches that of dentin, the stress transfer to the tooth–crown interface occurs more gradually, reducing stress concentrations that might cause cement failure or tooth fracture. However, if the modulus is too low, excessive crown flexure can lead to cement fatigue or progressive occlusal settling. The optimal moduli depend on specific clinical situations, remaining tooth structure, occlusal forces, and functional demands.

#### 4.1.3. Wear Behavior: The Long-Term Challenge

Wear resistance, adhesion, and fatigue mechanisms ultimately determine crown longevity for materials with adequate strength [60]. Ceramic crowns typically exhibit wear rates of 10–30 μm per year when opposed by natural enamel. Polymeric materials, unfortunately, demonstrate significantly higher wear rates, typically 50–150 μm per year, depending on formulation and opposing dentition [60]. The wear mechanism for polymeric crowns is complex and multifactorial [61]. Abrasive wear from food particles (particularly those containing mineral content) gradually removes material from occlusal surfaces. This polymer matrix preferentially wears away, leaving filler particles proud of the surface. These particles eventually deboned and detach, accelerating wear. Adhesive wear occurs when polymer chains bond temporarily to opposing surfaces, then fracture, pulling material away. Fatigue wear develops from repeated contact stresses that nucleate and propagate surface cracks. Chemical degradation from enzymes and acids in saliva can soften the surface polymer, making it more susceptible to mechanical wear. The molecular-level fatigue damage mechanisms (crack nucleation, crazing, and chain scission under cyclic loading) are described in detail in Section 4.1.4.

Chewing simulator studies provide accelerated wear testing by subjecting crowns to millions of loading cycles in artificial saliva or food slurries [60]. These studies consistently show that 3D-printed polymeric crowns exhibit 2–4 times higher wear than milled composite blocks and 5–10 times higher wear than ceramics. The difference traces to several factors: the lower filler content in printable formulations (40–50% vs. 70–80% in milled composites), the potential incomplete conversion leaving a softer matrix, and the residual porosity from the layered manufacturing process. Strategies to improve the wear resistance include maximizing the filler content while maintaining printability, optimizing the particle size distribution for better packing, using harder filler materials (alumina or zirconia rather than glass), improving the filler–matrix bonding through advanced silane treatments, and ensuring complete conversion through optimized curing protocols. Some experimental formulations incorporate self-lubricating fillers or surface treatments that reduce friction, showing promise in preliminary testing.

#### 4.1.4. Fatigue Endurance and Long-Term Reliability

Fatigue, the gradual damage from repeated loading, may be the most dangerous threat to crown longevity [62]. Unlike sudden overload failure, fatigue failure occurs slowly over thousands or millions of cycles, often at stress levels below the material’s maximum strength. For dental crowns experiencing 2000–3000 chewing cycles daily, fatigue endurance decides whether the restoration lasts months, years, or decades. Fatigue testing usually involves the cyclic loading of crown specimens at stress levels between 30–70% of the maximum strength while submerged in artificial saliva at 37 °C to mimic oral conditions. The load cycles are continued until failure or until reaching a set endpoint (often 1.2 million cycles, roughly 1–2 years of clinical use). A survival analysis then shows how many specimens survive at different cycle counts.

Research testing 3D-printed polymer crowns with such fatigue protocols show good short-term survival (>90% at 500, 500,000 cycles) but declining survival with more extended cycling [62]. By 1 million cycles, survival rates fall to 70–85%, depending on the material and processing. At 2 million cycles, fewer than 50% of specimens remain intact. For context, 2 million cycles roughly equal 2–3 years of clinical function, far less than the 10–15-year lifespan expected from ceramic crowns. The fatigue process involves crack formation at flaws or stress points, followed by gradual crack growth under repeated loads. Polymers are especially vulnerable because their lower modulus allows for larger strains, and their viscoelastic nature means energy is dissipated as heat instead of being stored elastically. Each loading cycle causes molecular damage, chain breaking, crazing, and microcrack formation, which build up over time.

Enhancing fatigue resistance involves addressing many factors: removing defects that can start cracks, improving material formulations for toughness, designing microstructures that redirect or stop cracks, and possibly adding stress-relief mechanisms that dissipate energy without damage. Some researchers are exploring layered materials, with stiff outer layers for wear resistance and tougher inner layers for crack stopping, as a way to boost fatigue performance.

### 4.2. Dimensional Accuracy: Achieving Clinical Fit

#### 4.2.1. Marginal Fit: The Critical Interface

The marginal fit, the gap between the crown margin and the tooth preparation finish line, critically influences clinical success through multiple mechanisms [63]. Excessive marginal gaps allow the microleakage of bacteria and oral fluids into the crown–tooth interface, promoting secondary caries that undermine the restoration. Large gaps also increase cement exposure to the oral environment, accelerating cement dissolution and potentially causing crown loosening. Beyond these biological concerns, visible marginal discrepancies compromise aesthetics, especially in the anterior region. Clinical studies have shown that marginal gaps exceeding 120 μm are linked to significantly higher failure rates [63]. Gaps in the 50–80 μm range are seen as clinically acceptable, while gaps below 50 μm are considered ideal. These standards come from decades of clinical research correlating the marginal quality with restoration longevity.

The evaluation of 3D-printed crowns using various measurement methods—including coordinate measuring machines, optical scanning, micro-CT imaging, and replica techniques—consistently reports marginal gaps in the 50–90 μm range [64]. This performance is comparable to milled crowns (usually 40–70 μm) and better than traditional crowns (often over 100 μm). Achieving a clinically acceptable marginal fit demonstrates that additive manufacturing can meet essential quality standards. However, maintaining a consistent marginal accuracy requires careful attention to several process parameters [65]. The print orientation greatly affects accuracy; crowns built with the margins parallel to the build layers tend to have a better marginal fit than those with the margins perpendicular to the layers. The support structure design impacts accuracy because the supports must be removed after printing, which can damage the delicate margin areas. Post-curing protocols may cause dimensional changes if thermal expansion during curing is not properly managed. Resin properties matter too; materials with higher polymerization shrinkage tend to produce tighter margins (which may be beneficial) but also generate higher internal stresses (which can be problematic).

#### 4.2.2. Internal Fit: Foundation for Retention

The internal fit, the gap between the crown interior and tooth preparation, affects both retention and stress distribution [66]. Insufficient internal space prevents the crown from seating fully, leading to open margins and a poor marginal fit. Excessive internal space requires thicker cement layers, which are more likely to dissolve and fail under fatigue. The ideal internal gap is generally considered to be 50–100 μm, enough to ensure complete seating and cement flow, yet thin enough to create a uniform cement layer. Measuring the internal fit is more challenging than measuring the marginal fit because the interior space is not directly accessible. Researchers use various methods: sectioning the crowns and measuring the gaps on cross-sections, using replica techniques where low-viscosity silicone fills the crown–tooth space and is then measured, or employing micro-CT scanning to create three-dimensional gap maps. Each method has its advantages and limitations.

Studies using micro-CT analysis, arguably the most comprehensive method, reveal that 3D-printed crowns achieve internal gaps similar to those of milled restorations when properly processed [66]. Gap distributions show expected patterns: smaller gaps at axial walls where parallel surfaces contact, and larger gaps at internal angles and line angles where the tooth preparation geometry creates space. Average gaps typically range from 80 to 120 μm, with standard deviations of 20 to 40 μm indicating a reasonably consistent fit.

Interestingly, research suggests that a slightly generous internal fit may actually benefit polymeric crowns. Because polymers are more flexible than ceramics, they can deform slightly during cementation to compensate for minor preparation irregularities. These flexibility characteristics might allow polymeric crowns to achieve intimate contact over a larger area than rigid ceramics, potentially improving retention. However, too much flexibility could also allow gradual creep deformation over time, which may compromise the long-term fit. This optimal balance remains a topic of ongoing research.

A comparative overview of the mechanical properties of 3D-printed polymeric crowns relative to conventional dental materials is presented in Table 3.

## 5. Biocompatibility and Clinical Integration

### 5.1. Biocompatibility: Safety at the Molecular Level

The biocompatibility evaluation of polymeric dental crown materials must address multiple safety dimensions as outlined by ISO 10993 (Biological Evaluation of Medical Devices), which provides the international standard framework for assessing cytotoxicity, sensitization, genotoxicity, and systemic toxicity [67]. Cytotoxicity data from in vitro cell culture studies (typically using L929 fibroblasts or human gingival fibroblasts per ISO 10993-5) confirm that fully post-cured 3D-printed dental resins generally demonstrate acceptable cytocompatibility [68]. However, insufficiently cured specimens release residual monomers and photoinitiators that are cytotoxic at concentrations achievable in the oral environment. Monomer elution quantification studies show that methacrylic acid, HEMA (2-hydroxyethyl methacrylate), UDMA (urethane dimethacrylate), and Bis-GMA (bisphenol A-glycidyl methacrylate) can leach from incompletely polymerized crowns; concentrations of HEMA above 3 mM have been shown to reduce the cell viability to below 70% [67,68]. Long-term degradation studies further reveal that ester bonds within the polymer backbone are susceptible to hydrolytic and enzymatic cleavage in the oral environment, releasing degradation products including methacrylic acid and glycerol dimethacrylate over periods of months to years. The relevance of BPA (bisphenol A) deserves specific mention: Bis-GMA, a common component of dental resins, may release trace amounts of BPA through hydrolysis, raising concerns about potential endocrine-disrupting effects [67]. While the current data suggest that the BPA release from post-cured dental resins is below the levels of toxicological concern in most formulations, the ongoing surveillance and selection of BPA-free resin systems are recommended where possible [69]. Taken together, these findings underscore that the biocompatibility of polymeric crowns is strongly dependent on complete polymerization conversion and rigorous post-curing protocols; inadequately processed restorations present a meaningfully greater biological risk.

The marginal fit, the gap between the crown margin and the tooth preparation finish line, critically influences clinical success through multiple mechanisms [63]. Excessive marginal gaps allow the microleakage of bacteria and oral fluids into the crown–tooth interface, promoting secondary caries that undermine the restoration. Large gaps also increase cement exposure to the oral environment, accelerating cement dissolution and potentially causing crown loosening. Beyond these biological concerns, visible marginal discrepancies compromise aesthetics, especially in the anterior region. The following paragraphs address the biocompatibility and safety aspects specific to polymeric dental crown materials.

### 5.2. Aesthetic Performance and Color Stability

Aesthetic performance covers several factors: initial color match, translucency, surface gloss, and long-term color stability [70,71,72]. For anterior crowns visible during smiling, aesthetic quality is crucial. Even posterior crowns need good aesthetics as patients increasingly reject visible metallic restorations. An initial color match for polymeric crowns is generally effective. Most 3D-printable resins come in various shades matching standard dental shade guides (e.g., VITA Classical shades A1–D4). These polymer matrices provide an intrinsic tooth-like appearance, and manufacturers add pigments and opacifiers to match specific shades. Achieving an excellent color match involves proper shade selection, usually comparing adjacent teeth with visual or spectrophotometric methods. However, the color stability over time is more challenging [73]. Studies tracking color changes in 3D-printed crowns exposed to coffee, tea, red wine, and other staining beverages show measurable shifts over weeks to months. The extent of these changes depends on the material formulation; more hydrophobic polymers resist staining better, whereas hydrophilic materials absorb staining molecules more easily. Six-month studies report color changes (ΔE values) of 2–4 units, which are noticeable but usually clinically acceptable. Longer-term data over several years would better predict clinical performance.

Natural enamel shows significant translucency, allowing the color of underlying dentin to impact the overall appearance. Early polymeric materials were opaque, resulting in a flat, lifeless look. Modern formulations use translucency-matching fillers and controlled pigmentation to better imitate natural optical properties. However, polymers still generally have less translucency than glass ceramics, limiting their use in highly aesthetic anterior restorations. Moreover, the surface characteristics greatly impact the appearance and function [74]. Smooth, glossy surfaces reflect light directly, creating an enamel-like shine. Rough surfaces scatter light diffusely, making them look dull. These as-printed surfaces typically need polishing to achieve an acceptable gloss. However, excessive polishing can remove surface layers and alter the color. Finding the right balance—being smooth enough for gloss and bacterial resistance without compromising dimensional accuracy—requires careful technique.

### 5.3. Patient Acceptance and Clinical Workflow Integration

Patient satisfaction ultimately determines clinical success regardless of technical performance [75]. Studies evaluating patient-reported outcomes for 3D-printed provisional crowns report high satisfaction across multiple dimensions: aesthetic appearance, comfort, function during eating, and overall experience. Patients especially appreciate the reduced treatment time; the same-visit delivery of provisional crowns eliminates the inconvenient temporary phase, during which ill-fitting temporaries can cause discomfort. The digital workflow itself enhances patient communication and engagement [76]. When patients see their crown designed on-screen in real time, they gain a better understanding and become more invested in the treatment. Virtual try-ins allow for an assessment of the aesthetics before fabrication, reducing remakes due to patient dissatisfaction. These technologies’ perceived sophistication also builds confidence; patients see digital crown fabrication as modern and precise compared to traditional methods. However, managing expectations remains crucial. Clinicians must clearly communicate that provisional polymeric crowns are temporary restorations with a limited lifespan. Patients sometimes form attachments to well-fitting provisionals and resist transitioning to final restorations. Clear communication during treatment planning helps prevent such issues.

## 6. Post-Processing: Optimizing Properties and Finishing

### 6.1. Post-Curing: Completing Polymerization

Post-curing is the most critical post-processing step for 3D-printed photopolymers [77]. This in-printer curing during printing results in only partial conversion, usually 60–80% depending on the material and exposure settings. This partial conversion is necessary for printing; complete conversion during exposure would make the layers too rigid for proper adhesion. Post-curing finishes the polymerization, maximizing conversion and greatly enhancing the mechanical properties and biocompatibility. The optimal post-curing protocols consider multiple factors [78,79]. The UV wavelength should match the photoinitiator’s absorption spectrum, usually 365 nm or 405 nm for dental materials. The exposure time must be long enough for full curing throughout the part; 20–30 min is sufficient for crowns up to 15 mm thick. The temperature during post-curing influences the cure speed; higher temperatures (60–80 °C) speed up the process and allow the conversion of unreactive species via thermal initiation. The atmosphere is also important; nitrogen or vacuum environments prevent oxygen inhibition, which can leave surfaces tacky and under-cured.

The consequences of inadequate post-curing are serious. The mechanical properties decline; the flexural strength can be 30–50% lower in uncured compared to properly post-cured parts. The dimensional stability worsens, and the residual reactive groups can slowly polymerize over time, causing shrinkage and distortion. The biocompatibility also drops; uncured monomers leach out more easily. The color stability decreases, and incomplete conversion makes the polymer more vulnerable to environmental degradation and yellowing. On the other hand, excessive post-curing can cause issues such as surface embrittlement and an increasing susceptibility to microcracks. Thermal degradation may occur if the temperature is too high or the exposure lasts too long. Dimensional changes due to thermal expansion during curing need to be considered; parts should be measured and adjusted after post-curing, not before.

### 6.2. Support Removal and Surface Finishing

Support structures, such as scaffolding printed to support overhanging features, must be carefully removed without damaging the crown [80]. Manual removal with pliers or scrapers requires skill to avoid gouging or breaking delicate areas. Excessive force can crack the crown, while inadequate removal leaves protrusions that interfere with fitting. Some manufacturers have developed specialized support materials that dissolve in solution, eliminating the need for manual removal. However, these sacrificial supports increase the material costs and processing complexity. Furthermore, the surface finishing transforms the as-printed part into a clinically acceptable restoration [81]. Progressive polishing with abrasive papers or rubber points removes layer lines and produces smooth surfaces. Diamond polishing pastes (typically 3-micron, 1-micron, and 0.25-micron progression) create a high-gloss finish. The goal is to achieve a surface roughness below 0.2 μm—the threshold above which bacterial adhesion significantly accelerates. However, excessive polishing can risk dimensional changes. Removing 50–100 μm from internal surfaces could compromise the fit. Careful technique emphasizes polishing external surfaces while preserving critical fitting areas. Some clinicians use selective polishing-extensive finishing on visible surfaces and minimal finishing on hidden areas.

### 6.3. Quality Assurance and Verification

Quality assurance protocols ensure consistent results across productions [82]. Digital verification using intraoral or laboratory scanners allows for the quantitative comparison of fabricated crowns to their design intent [83]. Superimposition analysis presents dimensional deviations as color-coded maps, typically showing deviations below 50 μm for well-controlled processes. These verifications should be performed routinely, not just during process validation, to detect the drift in equipment performance or material properties. Standardized testing protocols are still being developed by ISO and ADA technical committees [84]. The proposed standards address the material specifications, testing methodologies, and performance benchmarks. The adoption of these standards will help manufacturers demonstrate compliance through standardized testing and allow clinicians to compare products objectively. Currently, the lack of standards causes each manufacturer to use different tests, making comparisons difficult.

A comprehensive summary of post-processing parameters and their quantitative effects on material properties and clinical performance is presented in Table 4.

## 7. Current Challenges and Limitations Requiring Research

### 7.1. The Clinical Evidence Gap: Long-Term Data Deficit

Perhaps the most obvious limitation of 3D-printed polymeric crowns is the lack of long-term clinical data [85]. Most of the published research focuses on laboratory testing and short-term provisional uses. Clinical studies rarely go beyond 12–18 months, which is only a small part of the 10–15-year lifespan expected for permanent restorations [86]. This creates a paradoxical situation where the regulatory agencies hesitate to approve materials for permanent use without long-term data, but collecting such data requires clinical trials that need regulatory approval first.

The few longer-term studies available mainly focus on provisional applications, with reported 12-month survival rates of 85–95%. While promising, these figures do not predict how permanent restorations will perform. Provisional crowns endure different stresses; patients often avoid hard foods, knowing the temporary is less durable. Failure of a provisional crown is less serious than failure of a permanent restoration, and their success thresholds differ fundamentally.

For that, well-designed prospective clinical trials are urgently needed [87]. These studies should include appropriate control groups (like ceramic or Porcelain-Fused-to-Metal (PFM) crowns), standardized evaluation methods, enough participants for solid statistical results, and follow-up periods of at least 5 years (ideally 10+ years). Outcome measures should cover survival, biological issues (such as caries and periodontal problems), mechanical problems (like fractures and wear), and patient-reported outcomes. Only with such thorough evidence can it be determined whether polymeric crowns are suitable as permanent restorations.

### 7.2. Mechanical Property Limitations: The Strength Gap

The current 3D-printable polymers cannot match the mechanical properties of advanced ceramics [88]. Zirconia exhibits flexural strengths exceeding 900 MPa, roughly 6–8 times higher than those of printable polymers. Lithium disilicate reaches 400–500 MPa, still 3–4 times higher. These strength differences directly impact clinical performance; ceramics can withstand extreme loads that would fracture polymers. The fundamental challenge is improving the strength while maintaining printability [88]. Adding more filler enhances the strength but increases the viscosity, which may surpass printable limits. Using higher-strength polymers often involves higher melting temperatures (such as PEEK), requiring specialized equipment. Increasing the cross-linking density with multifunctional monomers raises the polymerization shrinkage and internal stress. Innovative strategies under investigation include nanocomposites with optimized particle size distributions for higher filler loading at an acceptable viscosity; surface-modified fillers that improve the matrix–filler bonding for better load transfer; reactive fillers that participate chemically in polymerization; gradient materials with stiff outer layers and tough cores; and biomimetic structures inspired by natural tooth enamel’s hierarchical architecture.

### 7.3. Lack of Standardization: Comparing Apples and Oranges

The lack of standardization in the materials, processes, and testing creates significant uncertainty [89]. Different manufacturers use varying resin formulations, each with its own proprietary composition. The printing parameters also differ widely; the layer thickness, exposure time, and post-curing protocols vary between systems. The testing methodologies lack consistency; some studies follow ISO standards, while others use ASTM or custom protocols. This heterogeneity makes it nearly impossible to compare the results across studies. For that, developing consensus standards is crucial for the field’s growth [89]. These standards should cover the material composition and specifications, manufacturing process parameters, physical and mechanical property testing, biocompatibility protocols, and clinical performance benchmarks. Such standards would give manufacturers clear targets, provide regulators with defined criteria for approval, and help clinicians make informed decisions through objective comparisons.

### 7.4. Regulatory Pathways: Navigating Approval Processes

Regulatory approval for 3D-printed medical devices presents distinct challenges [90]. The FDA has issued guidance documents recognizing additive manufacturing’s distinctive features: each printed device is technically unique, manufacturing occurs on demand rather than in batch production, and digital design files represent the “product” being controlled rather than physical inventory [91]. For dental crowns, the patient-specific nature further increases the regulatory complexity where each crown is a custom device designed for one individual. Traditional regulatory frameworks assume mass-produced, standardized products, in which batch testing validates quality. Consequently, the FDA approach focuses on validating the design process and printing process rather than each individual device. The design software must demonstrate that it consistently generates appropriate crown geometries, while printing systems must reliably reproduce digital designs. Although this process-validation approach is appropriate, it creates documentation burdens for manufacturers. A detailed analysis of the current challenges, their underlying root causes, impact on clinical translation, and corresponding potential solutions is presented in Table 5.

## 8. Future Directions and New Developments

### 8.1. Next-Generation Materials: Nanocomposites and Bioactive Formulations

The frontier of material development centers on nanocomposite formulations that incorporate nanoparticles to improve properties [92]. Unlike conventional microparticle fillers, nanoparticles (1–100 nm) can infiltrate between polymer chains, creating reinforcement at the molecular level. These enable simultaneous enhancements in strength, toughness, and wear resistance while maintaining an acceptable printing viscosity. Bioactive materials represent another exciting direction [93]. Imagine crowns that actively promote oral health instead of serving as passive restorations. Fluoride-releasing formulations could provide continuous remineralization, strengthening the surrounding tooth structure. Antimicrobial components could prevent biofilm formation and secondary caries. Calcium phosphate particles could stimulate mineralization at the crown–tooth interface, creating a chemical bond stronger than that provided by cement alone. Some experimental materials incorporate quaternary ammonium compounds or silver nanoparticles, showing potent antibacterial effects while maintaining biocompatibility. Smart materials that respond to environmental stimuli offer intriguing possibilities [94]. pH-responsive materials can detect acidic conditions indicating caries development and respond by releasing remineralizing agents. Temperature-sensitive polymers could expand during hot beverage consumption, sealing marginal gaps. Materials with self-healing capabilities could repair micro-cracks before they spread to cause failure. Although such concepts remain largely theoretical, preliminary laboratory demonstrations suggest their feasibility.

### 8.2. Artificial Intelligence and Machine Learning: Automating Design Excellence

Artificial intelligence, especially deep-learning algorithms, is set to revolutionize dental crown design [96]. Current AI uses in dentistry mainly focus on diagnosis, such as detecting cavities on radiographs, identifying periodontal bone loss, and segmenting anatomical structures. However, AI’s potential goes well beyond diagnosis into treatment planning and execution. For crown design, machine-learning systems can be trained on numerous successful cases to learn the optimal shape, occlusal contacts, and contour relationships [97]. A trained network could analyze a patient’s preparation scan, assess the surrounding and opposing teeth, and automatically create an optimized crown design within seconds. These designs would consider individual anatomical differences, occlusal loading patterns, aesthetic preferences, and even patient-specific factors like bruxism risk and dietary habits.

Integrating AI throughout the digital workflow promises significant improvements in efficiency and results [98]. Automated margin detection removes tedious manual outlining. Smart occlusal design guarantees proper contacts and movements. Predictive algorithms identify potential issues before fabrication. Quality control systems automatically inspect the created crowns, catching defects invisible to the naked eye. These advancements shift the clinician’s role from a technician (manually designing every detail) to a supervisor (approving AI suggestions and managing exceptions).

### 8.3. Four-Dimensional Printing: Adaptive Restorations

Four-dimensional printing extends 3D printing by incorporating the fourth dimension, time, through materials that change their properties or shape in response to stimuli. For dental applications, this could enable crowns that adapt to changing oral conditions. Shape-memory polymers could be printed in one configuration, then activated by the oral temperature to adopt a different shape, perhaps expanding to achieve intimate marginal adaptation. Materials could stiffen in response to loading, providing cushioning during light function but rigidity under heavy forces. While current 4D printing applications focus on biomedical devices such as self-expanding stents and drug-delivery systems, the underlying concepts translate well to dentistry. These challenge lies in identifying appropriate stimuli (temperature, pH, and pressure) and engineering materials with useful adaptive responses while maintaining biocompatibility and dimensional stability when adaptation is not desired.

### 8.4. Sustainability: Environmentally Conscious Manufacturing

Environmental sustainability is increasingly important in healthcare decision-making [95]. Traditional crown fabrication generates substantial waste, including impression materials, stone, wax, investment, and milling bur debris, which all accumulate. Ceramic sintering requires high-temperature furnaces consuming substantial energy. Digital workflows reduce some waste, but 3D printing still uses petroleum-based photopolymers with questionable end-of-life disposal options.

Bio-based polymers derived from renewable resources offer potential sustainability improvements. Methacrylates can be synthesized from plant oils rather than petroleum. Some manufacturers have developed resins with a 30–50% bio-based content that exhibit properties comparable to those of fully synthetic versions. Life cycle assessment studies help quantify environmental impacts across the entire product lifecycle, from raw material extraction through manufacturing, use, and eventual disposal [99]. Such analyses guide material selection toward more sustainable options.

## 9. Conclusions and Future Outlook

The three-dimensional printing of polymeric dental crowns stands at a fascinating inflection point, showing clear promise while facing significant challenges. This technology has unquestionably proven itself for provisional applications, where rapid fabrication, acceptable mechanical properties, and excellent dimensional accuracy combine to create compelling clinical value. These digital workflows’ efficiency, the elimination of traditional laboratory steps, and patient satisfaction with the reduced treatment time position 3D-printed provisional crowns as likely to become the standard of care in the coming years.

For permanent restorations, the path forward is less certain but not impossible where the current polymeric materials cannot match the mechanical properties, wear resistance, and demonstrated longevity of advanced ceramics. This performance gap relegates today’s polymers to provisional roles. However, the research directions outlined in this review, nanocomposites, bioactive formulations, gradient materials, and advanced processing techniques, show pathways toward clinically acceptable permanent restorations.

### 9.1. Current State: Achievements and Limitations

- Methacrylate-based resins have matured into reliable, well-characterized materials for provisional crown fabrication, offering an optimal combination of printability, mechanical properties, aesthetics, and biocompatibility for short-term applications.
- Vat photopolymerization technologies, particularly DLP and LCD systems, deliver the resolution, surface quality, and build speed necessary for clinical implementation, with a dimensional accuracy meeting or exceeding clinical requirements.
- High-performance polymers like PEEK exhibit mechanical properties superior to conventional composites, though still substantially below high-strength ceramics such as zirconia (flexural strength 300–1200 MPa vs. PEEK’s 150–200 MPa), and exceptional biocompatibility, though processing challenges and poor adhesion currently limit clinical adoption.
- Composite formulations successfully bridge the gap between pure polymers and ceramics. For mechanical properties, though, achieving optimal filler loading while maintaining printability remains an active research challenge.
- The digital workflow enabled by 3D printing offers significant advantages in efficiency, customization capability, and patient satisfaction compared to traditional manufacturing methods.

### 9.2. Critical Knowledge Gaps Requiring Research

- Long-term clinical evidence spanning 5–10 years is essentially nonexistent for permanent polymeric crown applications, creating a fundamental barrier to widespread clinical acceptance and regulatory approval.
- The mechanical properties of current printable polymers, particularly wear resistance and fatigue endurance, remain inferior to those of ceramics, limiting their use in permanent restorations until material innovations address these deficiencies.
- The standardization of materials, processing protocols, and testing methodologies is urgently needed to enable objective comparisons across materials and systems, guiding clinical decision-making and regulatory evaluation.
- Biocompatibility over extended periods, particularly as materials age and degrade in the oral environment, requires investigation through long-term animal studies and eventual clinical trials.
- Economic analyses comparing total treatment costs, including potential complications and remakes, are needed to objectively assess the value proposition of polymeric versus ceramic permanent restorations.

### 9.3. Pathways Forward: Promising Research Directions

- Advanced material formulations incorporating optimized nanocomposite structures, bioactive components, and novel toughening mechanisms show potential to significantly improve the mechanical properties while maintaining printability.
- Artificial intelligence and machine-learning applications promise to automate design optimization, predict clinical performance, and personalize restorations based on patient-specific risk factors and functional demands.
- Four-dimensional printing technologies enabling adaptive material responses could create restorations that adjust to changing oral conditions, potentially improving long-term clinical performance.
- Multi-material printing capabilities could enable gradient structures optimized for different functions, stiff occlusal surfaces for wear resistance, compliant cervical regions for stress distribution, and bioactive margins for caries resistance.
- The integration with broader digital dentistry workflows, including AI-assisted diagnosis, virtual treatment planning, and outcomes tracking, could position 3D-printed crowns within comprehensive, data-driven care models.

### 9.4. Final Perspective

- The evolution of polymeric 3D-printed dental crowns mirrors the broader trajectory of additive manufacturing: initial applications in prototyping and tooling, gradual expansion into functional end-use parts, and eventual acceptance as a mainstream production technology. Dentistry is further along this curve than many industries, with provisional restorations already established in clinical practice.
- Whether polymeric crowns will someday rival ceramics for permanent restorations remains an open question. This mechanical property gap is significant, and decades of clinical evidence support the longevity of ceramics. Polymers may never fully replace ceramics, but could carve out specific niches, patients with limited budgets, situations requiring rapid delivery, cases where ceramic aesthetics are unnecessary, or applications where polymer-specific advantages (like repairability or stress distribution) prove clinically valuable.
- More likely, the future involves diversification rather than replacement. Clinicians will have expanded treatment options spanning a spectrum from temporary to permanent, from economical to premium, and from ceramic to polymeric. Patient-specific factors, anatomy, function, finances, aesthetics, and systemic health will guide material selection from this broader palette. Advanced materials, such as bioactive nanocomposites, may create entirely new restoration categories that transcend current classifications.
- The technology is advancing rapidly. Materials science innovations occur continuously. Manufacturing capabilities improve year by year. Digital workflow integration deepens. Clinical evidence accumulates gradually. Regulatory frameworks adapt to new paradigms. In this dynamic field, today’s limitations may well become tomorrow’s historical footnotes. This field requires continued rigorous research, a honest assessment of the capabilities and limitations, and thoughtful clinical application guided by evidence rather than enthusiasm. With such a disciplined development, polymeric 3D-printed crowns will likely secure an important and enduring place in the prosthodontic armamentarium.

## Figures and Tables

**Table 1:** Comparison of polymeric material classes for 3D-printed dental crowns.

Material Class	Main Components	Primary Applications	Key Advantages	Main Limitations	Ref.
PMMA	Polymethyl methacrylate	Provisional crowns	Excellent aesthetics, ease of processing, and cost-effectiveness	Water sorption, plasticization, and limited long-term stability	[24,25,27]
Bis-acryl Composite Resins	Methacrylate monomers with inorganic fillers	Provisional crowns	Improved mechanical properties, adequate fracture resistance (>900 N)	Inferior to ceramics for permanent use	[28,29,30]
Composite Resins	Resin matrix with ceramic fillers	Provisional to semi-permanent crowns	Enhanced mechanical properties, good processability	Inferior wear resistance vs. milled composites, dependent on degree of conversion	[31,32,34,35]
PEEK	Polyetheretherketone	Permanent posterior crowns	Elastic modulus similar to bone, exceptional biocompatibility, and high strength	High melting temperature, requires specialized equipment, and adhesion challenges	[14,36,37,38,39]
Hybrid Resins (PICN)	Polymer-infiltrated ceramic networks	Permanent crowns	Improved fracture toughness vs. ceramics, interpenetrating network structure	Limited clinical data	[40,41,42]

**Table 2:** Comparison of additive manufacturing technologies for dental crown fabrication.

Technology	Working Principle	Resolution	Build Speed	Surface Quality	Material Options	Clinical Applications	Ref.
Stereolithography (SLA)	Focused laser scanning, layer-by-layer curing	High	Moderate	Excellent	Photopolymer resins	Provisional and definitive crowns, high accuracy (marginal gaps 50–90 μm)	[23,44,45,46]
Digital Light Processing (DLP)	Projects entire layer patterns simultaneously	High	Fast (faster than SLA)	Excellent	Photopolymer resins	Batch production of crowns, comparable accuracy to SLA	[47,48,49]
LCD Technology	Uses LCD screens as dynamic masks	Moderate to High	Fast	Good to Excellent	Photopolymer resins	Provisional crowns are a cost-effective alternative	[50,51]
Material Jetting	Multi-material droplet deposition	Very High	Slow	Excellent	Multi-material capability	Multi-colour crowns, gradient properties	[52,53,54]
Fused Filament Fabrication (FFF)	Thermoplastic extrusion	Low to Moderate	Moderate	Poor (visible layer lines)	Limited medical-grade filaments	Study models, surgical guides, and limited provisional use	[20,55,56]

**Table 3:** Mechanical properties comparison of 3D-printed polymeric crowns vs. conventional materials.

Property	3D Printed Polymers	Traditional Ceramics	Clinical Requirement	Test Method/Notes	References
Flexural Strength	80–120 MPa	300–1200 MPa	Varies by location	Depends on printing parameters and post-processing	[58,59]
Fracture Resistance	>900 N	>1500 N	>600 N (posterior), >300 N (anterior)	Sufficient for provisional applications	[22,59]
Marginal Gap	50–90 μm	40–80 μm	<120 μm	Clinically acceptable, influenced by print orientation	[63,64,65]
Internal Fit	Comparable to milled	Comparable to milled	Adequate for retention	Micro-CT analysis shows similar performance	[66]
Wear Resistance	Higher wear rates	Low wear rates	Long-term durability needed	Concern for permanent applications includes abrasive and fatigue components	[60,61]
Fatigue Performance	Adequate for provisional use	Excellent	Must survive cyclic loading	Limited durability for permanent applications under accelerated ageing	[62]

**Table 4:** Post-processing parameters and their quantitative effects on material properties and clinical performance.

Post-Processing Step	Parameters	Effect on Properties	Impact on Clinical Performance	Optimal Protocol	References
Post-Curing	UV wavelength: 405 nm; duration: 20 min	Optimizes mechanical properties, reduces residual monomer	Improved biocompatibility, enhanced strength	Temperature control during post-curing affects dimensional stability	[77,78,79]
Surface Finishing	Progressive polishing with diamond pastes	Reduces surface roughness to Ra < 0.2 μm	Decreases bacterial adhesion, improves aesthetics	Essential for bacterial retention prevention	[80,81]
Degree of Conversion	Influenced by post-curing time and temperature	Directly affects the mechanical properties and biocompatibility	Higher conversion = better clinical performance	Critical for long-term success	[35,69]
Quality Verification	Digital scanning and superimposition	Ensures dimensional accuracy	Quantitative assessment of fit	Standardized testing protocols under development	[82,83,84]

**Table 5:** Analysis of current challenges, root causes, impact on clinical translation, and potential solutions.

Challenge Category	Specific Issues	Current Limitations	Proposed Solutions/Future Directions	Impact on Clinical Adoption	Ref.
Clinical Evidence	Limited long-term data	Most studies < 12 months follow-up	Prospective clinical trials spanning 5–10 years are needed	Prevents permanent crown applications	[85,86,87]
Mechanical Properties	Inferior to ceramics	Strength and wear resistance are inadequate for permanent use	Nanocomposite resins, bioactive components, and advanced polymer development	Critical for permanent restoration approval	[33,88,92,93]
Standardization	Lack of consensus standards	Variability in materials, processes, and testing methods	ISO and ADA committees are developing protocols	Essential for regulatory approval	[84,89]
Regulatory Approval	Evolving FDA pathways	Specific requirements for dental applications under development	Clear guidelines needed for 3D-printed dental devices	Necessary for commercial adoption	[90,91]
Material Innovation	Need for improved polymers	Current materials are limited for permanent use	4D printing, nanocomposites, bio-based polymers, stimuli-responsive materials	Expands clinical applications	[53,94,95]
Design Optimization	Manual design limitations	Time-consuming, operator-dependent	AI-driven design, machine-learning algorithms for crown morphology and occlusion	Improves efficiency and outcomes	[96,97,98]
Sustainability	Environmental concerns	Petroleum-based polymers, waste generation	Bio-based polymers, life cycle assessment studies	Aligns with sustainable dental practice	[95,99]

## Data Availability

No new data were created or analyzed in this study. Data sharing is not applicable to this article.
